# Association among calf circumference, physical performance, and depression in the elderly Chinese population: a cross-sectional study

**DOI:** 10.1186/s12888-022-03925-z

**Published:** 2022-04-20

**Authors:** Jian-Yu Tan, Qing-Lian Zeng, Meng Ni, Ying-Xiao Zhang, Tian Qiu

**Affiliations:** 1 The First Affiliated Hospital of Chongqing Medical University, Youyi Road 1, Yuzhong district, Chongqing, 400016 China; 2 Department of Endocrinology, The First Affiliated Hospital of Chongqing Medical University, Youyi Road 1, Yuzhong district, Chongqing, 400016 China; 3 International Peace Maternity and Child Health Hospital, School of Medicine, Shanghai Jiao Tong University, Shanghai, China; 4 Department of Geriatrics, The First Affiliated Hospital of Chongqing Medical University, Youyi Road 1, Yuzhong district, Chongqing, 400016 China; 5 Department of Psychiatry, The First Affiliated Hospital of Chongqing Medical University, Youyi Road 1, Yuzhong district, Chongqing, 400016 China

**Keywords:** Depression, Sarcopenia, Muscle mass, Elderly, Calf circumference, Physical performance

## Abstract

**Background:**

Depression and sarcopenia are common diseases in the elderly population. However, the association between them is controversial. Based on the Chinese Longitudinal Healthy Longevity Survey (CLHLS) database, a cross-sectional study was conducted to explore the relationship of calf circumference and physical performance with depression.

**Methods:**

From the 8th wave of CLHLS conducted in 2018, data on calf circumference, physical performance, depressive symptoms, and demographic, socioeconomic, and health-related characteristics were collected. Multiple logistic regression was conducted to explore the impact of calf circumference, physical performance and their combination on depressive symptoms.

**Results:**

We enrolled a total of 12,227 participants aged 83.4 ± 11.0 years, including 5689 (46.5%) men and 6538 (53.5%) women. Patients with depression were more likely to have low calf circumference (2274 [68.2%] vs. 5406 [60.8%], *p*<0.001) and poor physical performance (3[0, 6] vs. 1[0, 4], *p*<0.001). A significant multiplicative interaction was found between calf circumference and physical performance in their effect on depression. After adjusting for confounding factors, multiple logistic regression showed that a significant inverse correlation persisted between physical performance and depressive symptoms in normal (odds ratio [OR] = 1.20, 95% confidence interval [CI]: 1.15–1.26, *p*<0.001) and low (OR = 1.14, 95% CI: 1.11–1.18, *p*<0.001) calf circumference group, while the association between calf circumference and depression disappeared. Participants with low calf circumference and poor physical performance were 2.21 times more likely to have depression than those with normal calf circumference and physical performance. All results were found to be robust in sensitivity analyses.

**Conclusions:**

Physical performance was significantly associated with depression in the elderly Chinese population. Attention should be paid to assess depressive symptoms in patients with poor physical performance.

**Supplementary Information:**

The online version contains supplementary material available at 10.1186/s12888-022-03925-z.

## Background

Depression, the most common type of mood disorder, is characterized by significant and persisting sadness [1]. According to a statistical analysis by the World Health Organization, depression is the leading cause of disability [2]. Depressive symptoms are common in later life, affecting approximately 10% of the elderly population [3].

Sarcopenia is characterized by age-related loss of skeletal muscle mass and function [4]. Its prevalence ranges from 5.5 to 25.7% in Asian countries [4] and may be underestimated in the elderly population [5]. The pathophysiology of sarcopenia includes oxidative stress, chronic inflammation, hormonal deficiency, physical inactivity, and malnutrition [6], which may also be involved in the pathogenesis of depression [7–11].

Exercise can improve mood in patients with depression and strengthen muscles, thus improving physical performance. In a randomized controlled trial, exercise intervention using a physical activity program positively affected the depressive status. Moreover, muscles are the largest group of tissues involved in exercise and endocrine metabolism. Therefore, many studies have investigated the relationship between sarcopenia and depression [12–17]. However, none of the studies considered a large sample size of the elderly Chinese population. Therefore, the aim of the present study was to investigate the association between calf circumference, physical performance and their combination on depression in the elderly Chinese population utilizing the Chinese Longitudinal Healthy Longevity Survey (CLHLS) database, a nationally representative sample.

## Methods

### Study population

Data from the 8th wave of the CLHLS conducted in 2018/2019 were utilized. Of all interviews, 82.1% were conducted in half of the randomly selected counties and cities from 22 provinces except the eight longevity areas, whereas 17.9% were conducted in the eight longevity areas and were relatively more in-depth [18]. The CLHLS attempted to interview all centenarians who voluntarily agreed to participate in the study in the sampled counties and cities. They adopted a targeted random-sample design to ensure representativeness. In the sampled counties, for every 3 centenarians, 4 participants aged 80–89, 4 participants aged 90–99, and 5 participants aged 65–79 were recruited based on predesignated age and sex. If there was no such matched person, a person will be recruited in neighboring county with same predesignated age and sex. In CLHLS, the trained staff had collected data from the elderly Chinese population through face-to-face interviews. The information included demographic, socioeconomic, lifestyle-related, and health-related characteristics. Detailed information of the CLHLS has been previously reported [19, 20].

**Fig. 1:**
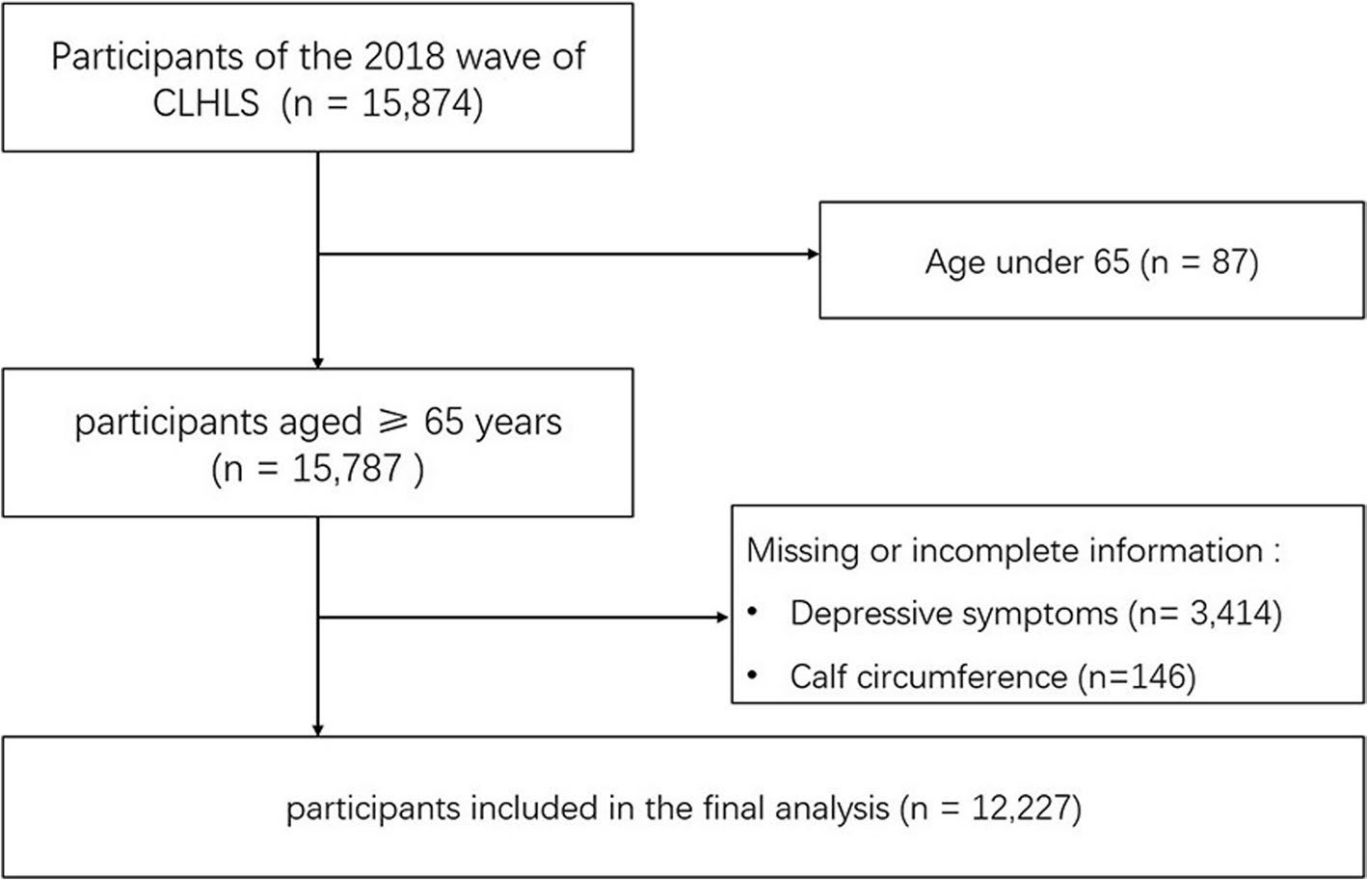
Flowchart of the selection of study population from participants of the Chinese Longitudinal Healthy Longevity Survey

We collected information on calf circumference and physical performance from the 2018 wave of the CLHLS and assessed depressive symptoms based on the Center for Epidemiologic Studies Depression Scale (CES-D). After excluding 3560 individuals with missing data on calf circumference or CES-D and 87 individuals aged below 65 years, a total of 12,227 participants were included in this study (Fig. 1).

### Measurements

The questionnaire in the 2018 wave of the CLHLS included calf circumference measured in cm. According to previous studies and the Asian Working Group for Sarcopenia (AWGS) 2019 consensus [4], calf circumference was used as a measure of muscle mass. The muscle mass is considered low when the calf circumference<34 cm in men and<33 cm in women [4, 21–23].

The questionnaire in the 2018 wave of the CLHLS assessed physical performance on a four-item scale. Muscle strength was assessed using the question “Are you able to lift 5 kg of weight?” Walking ability was assessed using the question “Are you able to walk 1 km?” The strength of the lower extremities was assessed using the question “Are you able to crouch and stand three times?” Finally, core strength was assessed using the question “How do you stand up after sitting in a chair?” The respondents were requested to choose from responses “without problem,” “with problem,” and “not able to,” which were assigned scores of 0, 1, and 2 points, respectively. The total score ranged from 0 to 8 points. A higher score indicated low physical performance. The internal consistency reliability using Cronbach’s α coefficient was 0.87. The principal component analysis generated one factor with eigenvalues ≥1, explaining 72% of the total variance. The 10-item CES-D-10 was used to assess depressive symptoms, as reported previously [24]. The options were “rarely,” “some days,” “occasionally,” and “most of the time,” which were assigned scores of 0, 1, 2, and 3 points, respectively. There were two positive questions: “Do you feel as happy as you did when you were young?” and “Are you full of hope for the future?” The results of these questions were reversely coded before summation. The total score ranged from 0 to 30 points. A higher score indicated greater severity of depressive symptoms. A score ≥ 10 in the CES-D-10 was considered to indicate depression, as validated by previous studies [25].

### Potential confounding factors

The accuracy of results was improved by adjusting for confounding factors, including demographic characteristics (age and sex), socioeconomic characteristics (residence, education, marital status, and retirement status), lifestyle- and health-related behaviors (smoking status, drinking status, activities of daily living [ADL]), social and leisure activity index, travel in the past two years, regular dietary intake of vegetables/fruits/meat/fish/milk products/food made from beans/eggs/nuts, regular tea drinking, and exercise), and health status (body mass index [BMI], cognitive function, and medical history).

The educational level was dichotomized according to the number of years of schooling (0 and ≥ 1 year). ADL ability was assessed on an eight-item scale. ADL disability was defined as the need for an assistant to perform one or more of the five activities (bathing, dressing, toileting, indoor transferring, and feeding) or being incontinent. The social and leisure activity index was calculated using questions on gardening, *tai chi chuan*, square dancing, reading, raising animals/pets, playing *mah-jongg*/cards, watching television/listening to the radio, and participating in social activities. The options were “almost every day,” “sometimes,” and “never,” which were assigned scores of 1, 2, and 3 points, respectively. The total score ranged from 8 to 24 points. A higher score indicated lack of social and leisurely activities.

The Chinese version of the 30-point Mini-Mental State Examination (MMSE) was used for cognitive function assessment. Cognitive impairment was defined as a total score < 24 on MMSE [19]. Medical history included a history of diabetes, heart disease, stroke, cancer.

### Statistical analysis

The data were statistically analyzed using the chi-square test, Student’s *t* test, or Mann–Whitney U test, as appropriate. Multiple imputations were used to fill in missing variables. No significant difference in baseline data was found before and after multiple imputations (data not shown). Multiple logistic regression was used to calculate the odds ratio (OR) of depressive symptoms for calf circumference and physical performance. In the multiplicative interaction analysis, low calf circumference, physical performance, and their product terms were included in regression analysis to investigate multiplicative interaction. In the additive interaction analysis, the Excel form provided by Mirjam J Knol et al. was used to calculate the RERI(relative excess risk due to interaction), AP(the attributable proportion due to interaction), and S(the synergy index) values [26]. Regarding significant interactions, analyses were stratified according to the type of calf circumference(normal and low calf circumference groups) and physical performance score(normal: 0 or 1 point; poor: 2–8 points). As 4909 (40.6%) and 1301 (10.8%) participants scored 0 and 1 point for physical performance, we merged them into one group for statistical analyses. Further, we merged those with 2–8 points into one group. To clarify the effects of calf circumference and physical performance on depression, a stepwise approach was used to adjust for different sets of confounding factors. Model 1 was adjusted for socioeconomic characteristics. Model 2 was additionally adjusted for lifestyle- and health-related behaviors. Model 3 was additionally adjusted for the health status.

Considering the interaction of calf circumference and physical performance, we combined them as two dichotomous variables and included them as a four dichotomous variables in multivariate logistic regression to evaluate the combined effect of these two factors on depression. The four categories were: normal calf circumference with normal physical performance; low calf circumference with normal physical performance; normal calf circumference with poor physical performance; and low calf circumference with poor physical performance.

Sensitivity analyses were conducted. First, multiple linear regression was conducted with calf circumference and depressive symptoms as continuous variables, considering interaction term. Second, multiple imputation was used to test the influence of missing data. Multiple logistic regression was conducted on the imputed datasets adjusted for model 3.

The data were analyzed using IBM SPSS Statistics version 20.0 for Windows (SPSS Inc., Chicago, IL). Two-tailed *p*-value <0.05 was considered statistical significance.

## Results

### General characteristics

This study involved 12,227 participants aged 83.4 ± 11.0 years, including 4904 participants aged 65–79 years and 7323 participants aged ≥80 years. The mean CES-D-10 score was 7.4 ± 4.5 (95% CI: 7.31–7.47). A CES-D-10 score ≥ 10, indicating depression, was found in 3335 (27.3%) participants (95% CI: 26.5–28.1%). Participants with depression were more likely to have low calf circumference (2274 [68.2%] vs. 5406 [60.8%], *p*<0.001) and poor physical performance(3 [0, 6] vs. 1 [0, 4], *p*<0.001; Table 1). Participants with depression were significantly older, predominantly female, less educated, more likely to be separated from their spouse or living alone, and retired without pension. They were more likely to have disability in ADL, and be less active in social and leisure activities. They traveled and exercised less and consumed an unhealthy diet. They had lower BMI, poor cognitive function, and more kinds of chronic diseases.

**Table 1:** Characteristics of the entire study population and subgroups based on the type of depression

Variables/Subgroups	Total sample	Depression (+)	Depression (−)	*p* value
Total sample, (n(%))	12,227	3335(27.3%)	8892(72.7%)	
Age (year, mean ± SD)	83.4 ± 11.0	84.7 ± 10.7	82.9 ± 11.1	<0.001
Female (n(%))	6538(53.5%)	2054(61.6%)	4484(50.4%)	<0.001
Rural residence (n(%))	5327(43.6%)	1477(44.3%)	3850(43.3%)	0.334
Educated (n(%))	6794(55.6%)	1506(45.2%)	5288(59.5%)	<0.001
Living with a spouse (n(%))	5469(44.7%)	1220(36.6%)	4249(47.8%)	<0.001
Retired with pension (n(%))	3534(28.9%)	775(23.2%)	2757(31%)	<0.001
Current smoker (n(%))	1991 (16.3%)	449(13.5%)	1542(17.3%)	<0.001
Current alcohol drinker (n(%))	1884(15.4%)	379(11.4%)	1505(16.9%)	<0.001
ADL disability (n(%))	2274(18.6%)	843 (25.3%)	1431 (16.1%)	<0.001
Social and leisure activity index (points, mean ± SD)	20.9 ± 2.4	21.6 ± 2.2	20.7 ± 2.4	<0.001
Have tourism in the past 2 years (n(%))	1671(13.7%)	279(8.4%)	1392(15.7%)	<0.001
Regular intake of (n(%))				
vegetable	8061(65.9%)	1848(55.4%)	6213(69.9%)	<0.001
fruit	2771(22.7%)	490(14.7%)	2281(25.7%)	<0.001
meat	5053(41.3%)	1217(36.5%)	3836(43.1%)	<0.001
fish	1170(9.6%)	279(8.4%)	891(10.0%)	<0.001
milk products	2913(23.8%)	627(18.9%)	2286(25.7%)	<0.001
food made from beans	1523(12.5%)	323(9.7%)	1200(13.5%)	<0.001
egg	4715(38.6%)	997(29.9%)	3718(41.8%)	<0.001
nut	818(6.7%)	131(3.9%)	687(7.7%)	<0.001
tea	2236(18.3%)	426(12.8%)	1810(20.4%)	<0.001
Regular exercise (n(%))	4230(34.6%)	757(22.7%)	3473(39.1%)	<0.001
BMI (kg/m^2^, mean ± SD)	22.4 ± 8.0	21.8 ± 9.0	22,7 ± 7.5	<0.001
Low calf circumference (n(%))	7680(62.8%)	2274(68,2%)	5406(60.8%)	<0.001
Physical performance index (points, mean ± SD)	1(0,4)	3(0,6)	1(0,4)	<0.001
Cognitive impairment (n(%))	2718(22.2%)	1044(31.3%)	1674(18.8%)	<0.001
History of disease (n(%))				
Diabetes	1381(11.3%)	384(13.2%)	844(10.5%)	<0.001
Heart disease	2314(18.9%)	678(23.1%)	1394(17.2%)	<0.001
Stroke	1433(11.7%)	438 (15.1%)	830(10.3%)	<0.001
Cancer	1197(9.8%)	320(9.6%)	877(9.9%)	0.684

*ADL* activities of daily living, *BMI* body mass index

Data are expressed as mean ± standard deviation (effective sample size), median (25 and 75% quartiles, effective sample size), and number (%), as appropriate

The *p*-values of sex, living residence, education level, marital status, retirement status, smoking status, drinking status, activities of daily living, travel, dietary habit, exercise habit, calf circumference, cognitive function, medical history were calculated by using the chi-square test; The *p*-values of age, social and leisure activity index, BMI were calculated by using the Student’s *t*-test; The *p*-value of physical performance index was calculated using the Mann–Whitney U test. All *p*-values were < 0.001, except for rural residence (*p* = 0.334) and history of cancer (*p* = 0.684)

### Association of combined calf circumference and physical performance with depressive symptoms

To verify the possible interaction between calf circumference and physical performance, the interaction terms of the two factors were included in the regression model. After adjusting for confounding factors, the interaction term remained statistically significant(OR = 0.94, 95% CI: 0.91–0.98, *p*< 0.05), implying that multiplicative interaction exists between calf circumference and physical performance (Table 2) [27]. RERI(95%CI) = −0.03(−0.09–0.04), AP(95%CI) = −0.02(−0.06–0.02), S(95%) = 0.95(0.85–1.08), which means there is no significant additive interaction between low calf circumference and physical performance [26–28]. Stratified by the type of calf circumference, physical performance showed a significant association with depression after adjusting for confounding factors. In the normal calf circumference group, with every 1-point increase in physical performance, the risk of depression increased by 20% (OR = 1.20, 95% CI: 1.15–1.26, *p*<0.001). In the low calf circumference group, with every 1-point increase in physical performance, the risk of depression increased by 14% (OR = 1.14, 95% CI: 1.11–1.18, *p*<0.001). Conversely, in the normal physical performance group(score 0 or 1 point), the association between calf circumference and depression attenuated after adjusting for confounding factors (OR = 1.13, 95% CI: 0.98–1.30, *p* = 0.06). No significant association was found in the poor physical performance group between calf circumference and depression.

**Table 2:** Multiple logistic regression analysis of the association of calf circumference and physical performance with depression

	Unadjusted		Adjusted for Model 1		Adjusted for Model 2		Adjusted for Model 3	
	OR	95%CI	OR	95%CI	OR	95%CI	OR	95%CI
Low calf circumference	1.36^***^	1.20–1.53	1.36^***^	1.20–1.54	1.23^**^	1.09–1.40	1.23^**^	1.08–1.40
Physical performance	1.24^***^	1.20–1.27	1.28^***^	1.24–1.32	1.23^***^	1.19–1.28	1.21^***^	1.17–1.25
Low calf circumference by physical performance	0.93^***^	0.90–0.97	0.93^***^	0.90–0.96	0.94^***^	0.91–0.97	0.94^**^	0.91–0.98
Calf circumference(A group)								
Physical performance	1.24^***^	1.20–1.27	1.28^***^	1.23–1.33	1.23^***^	1.18–1.28	1.20^***^	1.15–1.26
Calf circumference(B group)								
Physical performance	1.16^***^	1.14–1.18	1.19^***^	1.17–1.22	1.16^***^	1.13–1.19	1.14^***^	1.11–1.18
Physical performance(A group)								
Low calf circumference	1.30^***^	1.15–1.49	1.21^**^	1.06–1.39	1.12	0.97–1.28	1.13^*^	0.98–1.30
Physical performance(B group)								
Low calf circumference	1.10	0.97–1.24	1.09	0.96–1.24	1.01	0.89–1.15	1.02	0.89–1.16

^*^p-value <0.1; ^**^p-value <0.05; ^***^p-value <0.001

Calf circumference (A group: ≥ 34 cm in men or ≥ 33 cm in women; B group <34 cm in men or < 33 cm in women)

Physical performance (A group: 0 or 1 point; B group: 2–8 points)

Model 1 has been adjusted for socioeconomic characteristics (age, sex, rural residential area, years of education, marital status, and retirement status)

Model 2 has been additionally adjusted for lifestyle- and health-related behaviors (alcohol and cigarettes consumption, ability for activities of daily living, social and leisure activity index, regular dietary intake of vegetables/fruits/meat/fish/milk products/food made from beans/eggs/nuts, regular tea drinking, and exercise)

Model 3 has been additionally adjusted for the health status (body mass index, cognitive function, a history of some diseases, and the number of chronic diseases)

We explored patterns of the joint effect of calf circumference and physical performance on depressive symptoms. Table 3 shows the OR estimates for the strata as defined by the four pairs formed from two categories of calf circumference and two categories of physical performance. Participants with low calf circumference and poor physical performance were 2.21 times more likely to have depression than those with normal calf circumference and physical performance. The highest depression rate, at 36.9%, was found in the group with low calf circumference and poor physical performance. Trend test showed that the risk of depression increased as the number of factors increased. (*p* for trend< 0.001).

**Table 3:** Combined effect of calf circumference and physical performance on depressive symptoms

Calf circumference	Physical performance	Depression rate (%)	Unadjusted		Adjusted for model 3	
			OR	95%CI	OR	95% CI
–	–	16.5%	1.00		1.00	
+	–	20.7%	1.32^***^	1.16–1.50	1.17^**^	1.02–1.34
–	+	34.5%	2.66^***^	2.31–3.06	2.16^***^	1.84–2.54
+	+	36.9%	2.96^***^	2.63–3.33	2.21^***^	1.90–2.57
*p* for trend			<0.001		<0.001	

^*^p-value <0.1; ^**^p-value <0.05; ^***^p-value <0.001

Calf circumference (−: ≥ 34 cm in men and ≥ 33 cm in women; +: < 34 cm in men and < 33 cm in women)

Physical performance (−: 0 or 1 point; +: 2–8 points)

Model 3 has been adjusted for age, sex, rural residential area, years of education, marital status, retirement status, alcohol consumption, smoking status, ability for activities of daily living, social and leisure activity index, regular dietary intake of vegetables/fruits/meat/fish/milk products/food made from beans/eggs/nuts, regular tea drinking, exercise, body mass index, cognitive function, a history of some diseases, and the number of chronic diseases

### Sensitivity analysis

In the sensitivity analysis, we conducted multiple linear regression to test the robustness of the results. Depressive symptoms expressed as scores and calf circumference as cm. Because of the statistical significance of the interaction term (*p* = 0.01). We conducted a stratified analysis. Physical performance showed a consistent association with depression in both normal and low circumference group after adjusting for model 3. However, the impact of calf circumference attenuated after adjusting for confounding factors in both groups of physical performance (scores: 0 or 1 point and 2–8 points; Supplementary Table 1). The results of multiple logistic regression after multiple imputations as sensitivity analysis were consistent with the final results.

## Discussion

Utilizing a nationally representative large-scale survey of the elderly (aged ≥65 years) Chinese population, a cross-sectional study was conducted to investigate the relationship of calf circumference and physical performance with depression. The association between physical performance and depression was found to be pronounced, consistent with previous studies [29, 30]. Physical inactivity was a common risk factor for sarcopenia and depression and could mediate the relationship between them [31]. Furthermore, skeletal muscle and the brain show crosstalk [32]. Contracting skeletal muscles secrete neurotrophic factors, which play a role in mood. Neurotrophins, such as brain-derived neurotrophic factor and neurotrophin-3, which promote neuronal differentiation, survival, and synaptic potentiation, are produced by both the brain and skeletal muscle and are associated with mood and muscle regeneration [33]. This may be the potential mechanism underlying the association between sarcopenia and mental illness, as decreased neurotrophic support of the brain is associated with depression and anxiety [34]. Chronic low-grade inflammation and oxidative stress, which are associated with both depression and sarcopenia, may be another possible mechanism [7, 35]. These studies imply that the relationship between sarcopenia and depression may be mediated by physical activity, brain plasticity and inflammation. Potential intervention of these factors might not only relieve depression but also improve sarcopenia.

In this study, people with larger calf circumferences were less likely to have depressive symptoms. Further, after adjusting for potential confounding factors, the impact of calf circumference attenuated, consistent with previous studies [30, 36]. A European consensus indicates that age-related changes in fat deposits and loss of skin elasticity can lead to estimation errors of the calf circumference in the elderly population [37]. Therefore, calf circumference may not be a good predictor of muscle mass in that population. Individuals may have developed sarcopenic obesity because of lack of exercise, which can result in a reduced predictive value of the calf circumference for muscle mass.

The interaction analysis showed a multiplicative interaction between low calf circumference and physical performance. Considering no addictive interaction was found, the biological effect of joint exposure needs to be elucidated in further study. Subsequently, in the joint effect analysis, we found that people with both low calf circumference and the poor physical performance had the highest morbidity of depression. They were 2.21 times more likely to have depression than those with normal calf circumference and physical performance. These analyses suggest that low calf circumference and physical performance may work in a synergistic way to increase the risk of depression in older adults. The above results suggest we should provide professional medical services on time for susceptible groups. Some similar previous studies have been conducted in ethnic groups other than the Chinese. An English study showed that reduction in grip strength is associated with a higher risk of depressive symptoms in participants with obesity [16]. A Japanese cross-sectional study found a significant association between sarcopenia and depression in elderly male patients with diabetes, measured by “Strength, assistance with walking, rising from a chair, climbing stairs, and falls” (SARC-F) and CES-D questionnaires [38]. A Korean study found that the coexistence of low muscle mass and low muscle function(assessed using dual-energy X-ray absorptiometry scanning and sit-to-stand mean power based on a 30 s chair stand test) is significantly associated with an increased risk of depressive symptoms [39]. Because of the complexity of sarcopenia diagnosis, many studies have shown a correlation with one component of its diagnostic criteria, insufficient to prove a link between muscle condition (muscle mass, muscle strength, or physical performance) and depression. More prospective and large-scale studies are required to clarify the relationship between muscle condition and depression.

The advantage of our study was the use of a large sample size of the elderly Chinese population. To obtain robust results, we adjusted for many confounding factors. However, the study has some limitations. First, we selected four questions in the questionnaire to evaluate physical performance in reference to SARC-F, which was recommended to screening sarcopenia by AWGS 2019 Consensus [3]. However, the evaluation efficiency of the questionnaire composed of these four questions has not been tested. Therefore, the results of the present study may lack universality. Second, we excluded 3414 participants because of missing data on CES-D. Comparing the baseline characteristics of the excluded participants with the included participants, revealed that the data were not missing completely at random, which might influenced the results. Therefore, we used multiple imputation to evaluate the impact of missing data on the results. Third, we evaluated physical performance by a subjective questionnaire, which might be inaccurate because of the depressive symptoms of the participants, making them choose more negative answers rather than actual answers. Or we can’t rule out the possibility that their physical performance is reduced due to depression-induced low physical activity. Causality is difficult to investigate because of our cross-sectional study design. Prospective studies are required to address this limitation.

## Conclusions

Depression and sarcopenia are common diseases in the elderly population. This study showed a strong association between physical performance and depression, but no significant association between calf circumference and depressive symptoms. Future studies should focus on the comorbidities of depression and sarcopenia in the elderly population.

## Supplementary Information

**Additional file 1: Supplementary table 1.** Sensitivity analysis of the multiple linear regression for the association of calf circumference and physical performance with depressive symptoms. **Supplementary table 2.** Sensitivity analysis of the multiple logistic regression for the association of calf circumference and physical performance with depressive symptoms after multiple imputation.

## Data Availability

The data that support the findings of this study are available in https://opendata.pku.edu.cn/dataverse/CHADS [40].

## Abbreviations

CLHLS: Chinese Longitudinal Healthy Longevity Survey
OR: Odds ratio
CI: Confidence interval
CES-D: Center for Epidemiologic Studies Depression Scale
AWGS: Asian Working Group for Sarcopenia
ADL: Activities of daily living
BMI: Body mass index
MMSE: Mini-Mental State Examination
RERI: Relative Excess Risk due to Interaction
AP: Attributable Proportion
S: the Synergy index
