# Supplementary material for: Association among calf circumference, physical performance, and depression in the elderly Chinese population: a cross-sectional study
Source: BMC Psychiatry. 2022 Apr 20;22:278. doi: 10.1186/s12888-022-03925-z (PMC9020001; doi:10.1186/s12888-022-03925-z)
Supplement: Supplementary file 1 — Additional file 1: Supplementary table 1. Sensitivity analysis of the multiple linear regression for the association of calf circumference and physical performance with depressive symptoms. Supplementary table 2. Sensitivity analysis of the multiple logistic regression for the association of calf circumference and physical performance with depressive symptoms after multiple imputation. [file 12888_2022_3925_MOESM1_ESM.docx]

**Supplementary table 1. Sensitivity analysis of the multiple linear regression for the association of calf circumference and physical performance with depressive symptoms**

|  | Unadjusted | | | Adjusted for Model 3 | | |
| --- | --- | --- | --- | --- | --- | --- |
|  | Unstandardized β | Standardized β | *p* | Unstandardized β | Standardized β | *p* |
| Calf circumference (A group) |  |  |  |  |  |  |
| Physical performance | 0.52 | 0.27 | ＜0.001 | 0.42 | 0.22 | ＜0.001 |
| Calf circumference (B group) |  |  |  |  |  |  |
| Physical performance | 0.38 | 0.24 | ＜0.001 | 0.34 | 0.21 | ＜0.001 |
| Physical performance (A group) |  |  |  |  |  |  |
| Calf circumference | -0.04 | -0.06 | ＜0.001 | -0.01 | -0.02 | 0.201 |
| Physical performance (B group) |  |  |  |  |  |  |
| Calf circumference | -0.00 | -0.00 | 0.761 | 0.01 | 0.01 | 0.334 |

**Calf circumference and depressive symptoms are included in the regression equation as continuous variables.**

**Calf circumference (A group: ≥ 34 cm in men and ≥ 33 cm in women; B group: < 34 cm in men and < 33 cm in women)**

**Physical performance (A group: 0 or 1 point; B group: 2–8 points)**

**Model 3 has been adjusted for age, sex, rural residential area, years of education, marital status, retirement status, alcohol consumption, smoking status, ability for activities of daily living, social and leisure activity index,** **regular dietary intake of vegetables/fruits/meat/fish/milk products/food made from beans/eggs/nuts, regular tea drinking, exercise, body mass index, cognitive function, a history of some diseases, and the number of chronic diseases**

**Supplementary table 2. Sensitivity analysis of the multiple logistic regression for the association of calf circumference and physical performance with depressive symptoms after multiple imputation**

|  | Unadjusted | | Adjusted for model3 | |
| --- | --- | --- | --- | --- |
|  | OR | 95% CI | OR | 95% CI |
| Calf circumference (A group) |  |  |  |  |
| Physical performance | 1.24^***^ | 1.21-1.27 | 1.18^***^ | 1.09-1.27 |
| Calf circumference (B group) |  |  |  |  |
| Physical performance | 1.16^***^ | 1.14-1.18 | 1.15^***^ | 1.10-1.21 |
| Physical performance (A group) |  |  |  |  |
| Calf circumference | 1.35^***^ | 1.19-1.52 | 1.11 | 0.87-1.41 |
| Physical performance (B group) |  |  |  |  |
| Calf circumference | 1.11^*^ | 0.99-1.23 | 1.00 | 0.80-1.26 |

**^*^*p*-value < 0.1; ^**^*p*-value < 0.05; ^***^*p*-value < 0.001**

**Calf circumference (A group: ≥ 34 cm in men and ≥ 33 cm in women; B group: < 34 cm in men and < 33 cm in women)**

**Physical performance (A group: 0 or 1 point; B group: 2–8 points)**

**Model 3 has been adjusted for age, sex, rural residential area, years of education, marital status, retirement status, alcohol consumption, smoking status, ability for activities of daily living, social and leisure activity index,** **regular dietary intake of vegetables/fruits/meat/fish/milk products/food made from beans/eggs/nuts, regular tea drinking, exercise, body mass index, cognitive function, a history of some diseases, and the number of chronic diseases.**
